# Identification of a Novel Theranostic Signature of Metabolic and Immune-Inflammatory Dysregulation in Myocardial Infarction, and the Potential Therapeutic Properties of Ovatodiolide, a Diterpenoid Derivative

**DOI:** 10.3390/ijms23031281

**Published:** 2022-01-24

**Authors:** Alexander T. H. Wu, Bashir Lawal, Yew-Min Tzeng, Chun-Che Shih, Chun-Ming Shih

**Affiliations:** 1 The Ph.D. Program of Translational Medicine, College of Medical Science and Technology, Taipei Medical University, Taipei 11031, Taiwan; chaw1211@tmu.edu.tw; 2 Clinical Research Center, Taipei Medical University Hospital, Taipei Medical University, Taipei 11031, Taiwan; 3 TMU Research Center of Cancer Translational Medicine, Taipei Medical University, Taipei 11031, Taiwan; 4 Graduate Institute of Medical Sciences, National Defense Medical Center, Taipei 11490, Taiwan; 5 Taipei Heart Institute, Taipei Medical University, Taipei 11031, Taiwan; ccshih0603@tmu.edu.tw; 6 Ph.D. Program for Cancer Molecular Biology and Drug Discovery, College of Medical Science and Technology, Taipei Medical University and Academia Sinica, Taipei 11031, Taiwan; d621108004@tmu.edu.tw; 7 Graduate Institute for Cancer Biology & Drug Discovery, College of Medical Science and Technology, Taipei Medical University, Taipei 11031, Taiwan; 8 Department of Life Science, National Taitung University, Taitung 95092, Taiwan; president@nttu.edu.tw; 9 Division of Cardiovascular Surgery, Department of Surgery, Wan Fang Hospital, Taipei Medical University, Taipei 11696, Taiwan; 10 Department of Surgery, School of Medicine, College of Medicine, Taipei Medical University, Taipei 11031, Taiwan; 11 Institute of Clinical Medicine, National Yang Ming Chiao Tung University, Taipei 11221, Taiwan; 12 Department of Internal Medicine, School of Medicine, College of Medicine, Taipei Medical University, Taipei 11031, Taiwan; 13 Division of Cardiology, Department of Internal Medicine, Taipei Medical University Hospital, Taipei 11031, Taiwan

**Keywords:** myocardial infarction, theranostic, inflammatory and metabolic dysregulation, miRNA, ovatodiolide, DEG

## Abstract

Myocardial infarction (MI) is a multifactorial global disease, recognized as one of the leading causes of cardiovascular morbidity and mortality. Timely and correct diagnoses and effective treatments could significantly reduce incidence of complications and improve patient prognoses. In this study, seven unconventional differentially expressed genes (DEGs) (MAN2A2, TNFRSF12A, SPP1, CSNK1D, PLAUR, PFKFB3, and CXCL16, collectively termed the MTSCPPC signature) were identified through integrating DEGs from six MI microarray datasets. The pathological and theranostic roles of the MTSCPPC signature in MI were subsequently analyzed. We evaluated interactions of the MTSCPPC signature with ovatodiolide, a bioactive compound isolated from *Anisomeles indica* (L.) Kuntze, using in silico molecular docking tools and compared it to specific inhibitors of the members of the MTSCPPC signature. Single-cell transcriptomic analysis of the public databases revealed high expression levels of the MTSCPPC signature in immune cells of adult human hearts during an MI event. The MTSCPPC signature was significantly associated with the cytokine–cytokine receptor interactions, chemokine signaling, immune and inflammatory responses, and metabolic dysregulation in MI. Analysis of a micro (mi)RNA regulatory network of the MTSCPPC signature suggested post-transcriptional activation and the roles of miRNAs in the pathology of MI. Our molecular docking analysis suggested a higher potential for ovatodiolide to target MAN2A2, CSNK1D, and TNFRSF12A. Collectively, the results derived from the present study further advance our understanding of the complex regulatory mechanisms of MI and provide a potential MI theranostic signature with ovatodiolide as a therapeutic candidate.

## 1. Introduction

Myocardial infarction (MI) is a multifactorial global disease, recognized as one of the leading causes of cardiovascular morbidity and mortality in both men and women [1]. It is caused by the partial or complete occlusion of a coronary artery, which blocks the supply of oxygen and nutrients to the myocardium, leading to myocardial cell death [2]. MI may occur transiently or in a disastrous manner that could lead to hemodynamic valve deterioration and sudden death [2,3]. Risk factors for MI are classified into non-modifiable, modifiable, and emerging risk factors [4]. Non-modifiable risk factors include age, gender, and family history; modifiable risk factors are related to one’s lifestyle (such as a poor diet, smoking, alcohol intake, a sedentary lifestyle, etc.), dyslipidemia, diabetes, hypertension, and metabolic disorders, while emerging risk factors are coronary artery calcification (CAC), C-reactive protein (CRP), homocysteine, fibrinogen, and lipoproteins [4].

Timely and correct diagnoses and effective treatments can significantly reduce incidence of complications and improve the prognosis of patients with MI [5]. The current diagnostic methods for MI involve a physical examination, clinical history, cardiac markers, electrocardiography, and evidence of myocardial ischemia. The levels of cardiac-specific troponins T (cTnT) and I (cTnI) are the preferred biomarkers for the evaluation of myocardial injury and high-sensitivity (hs)-cTn assays are recommended for routine clinical use [4,6]. Other less important serum biomarkers include creatine kinase (CK), MB isoforms of creatine (CK-MB), and myoglobin [4]. However, the reliability of these biomarkers is limited by their inadequate specificity and sensitivity [7,8,9,10], which can lead to false diagnostic outcomes.

Although myocardial injury (defined by an elevated cTn value) is a prerequisite for the diagnosis of MI, the presence or absence of nonischemic myocardial injury must be confirmed in order to establish a reliable diagnosis [11]. Hence, the current Fourth Universal Definition of Myocardial Infarction Consensus Document defined clinical criteria for MI as the presence of acute myocardial injury detected by elevated cTn values above the 99th percentile upper reference limit (URL) in the setting of evidence of acute myocardial ischemia [12].

Despite increased understanding of the causes and treatments of MI, high rates of morbidity and mortality still remain [13]. Although great progress has been made with percutaneous coronary intervention (PCI), which provided good treatment outcomes in MI Patients presenting with ST-segment elevation [14] as well as in patients presenting without persistent ST-segment elevation [15], there is an urgent need to find more sensitive and specific genetic markers for early diagnoses and for the development of novel targeted therapies for better prognoses of patients with MI [16].

Bioinformatics or in silico analysis of clinical data, novel targets, and drug candidate identification are indispensable parts of modern research strategies for disease prevention and treatment [17]. Targets for drug discovery could be biological pathways, abnormal molecular phenotypes, essential nodes of biological network or molecular functions, and disease-related microRNAs, genes, or proteins [18,19,20,21,22,23]. In the present study, differentially expressed genes (DEGs) from six MI microarray datasets were integrated to identify overlapping DEGs, and the pathological and theranostic roles of these DEGs in MI were subsequently analyzed. The pathological mechanisms of MI involved the interaction of several regulatory networks involving cytokine–cytokine receptors, chemokine signaling, immune and inflammatory responses, and metabolic dysregulation. Hence, targeted modulation of immune, inflammatory, and metabolic pathways could yield a novel and effective therapeutic approach for preventing and treating MI.

Natural products are rich sources of health-promoting bioactive compounds for treating numerous diseases [24,25,26,27]. Ovatodiolide is a bioactive compound isolated from a Taiwanese plant, *Anisomeles indica* (L.) Kuntze, that is commonly and traditionally used for treating inflammation-associated diseases [28]. The therapeutic properties of ovatodiolide against various diseases are well described in the literature [28,29,30,31,32,33,34]. It is also an important modulator of inflammatory responses [35] and can serve as a potential immunotherapeutic agent [36]. However, despite the extensive medicinal use of the plant, there are no reports available on its use for MI. The use of molecular docking has greatly aided drug development by providing information related to interactions of therapeutic agents with molecular targets of disease [37,38]. Hence, we evaluated interactions of ovatodiolide with DEGs using in silico molecular docking studies. Collectively, results from the present study have aided our understanding of the complex regulatory mechanisms of MI and provide a promising approach for MI theranostic markers. We await future validation and investigation of the therapeutic role of ovatodiolide in experimental and clinical MI.

## 2. Results

### 2.1. Identification of MAN2A2/TNFRSF12A/SPP1/CSNK1D/PLAUR/PFKFB3/CXCL16 as a Novel Pathological Signature of Myocardial Infarction

**Figure 1:**
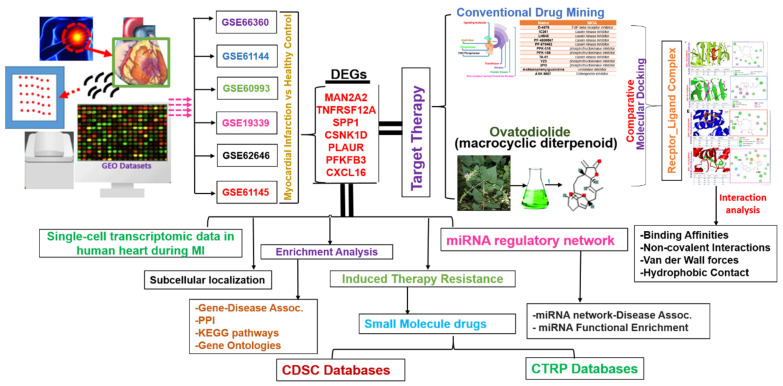
Schematic representation of the study workflow for the identification of the theranostic signature of metabolic and immune-inflammatory dysregulation with myocardial infarction and the potential therapeutic function of ovatodiolide, a macrocyclic diterpenoid.

**Figure 2:**
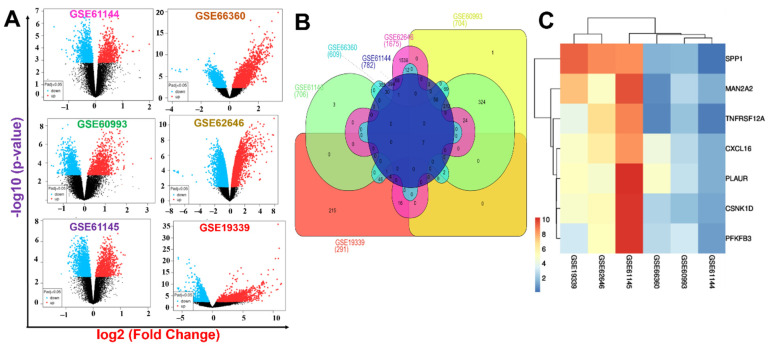
Identification of the potential genes related to myocardial infarction (MI). (**A**) Volcano plots of differentially expressed genes (DEGs) in the microarray datasets between MI patients and healthy controls. (**B**) Venn diagram showing the numbers of DEGs in each dataset and intersections of the DEGs from all MI datasets. (**C**) Heat maps of the intersected upregulated DEGs from the six datasets. Seven upregulated DEGs (MAN2A2, TNFRSF12A, SPP1, CSNK1D, PLAUR, PFKFB3, and CXCL16) the MTSCPPC signature associated with MI were identified.

To study deregulated genes associated with MI, we analyzed DEGs between healthy and MI cohorts. The workflow of the entire study is summarized in Figure 1. DEGs from six GEO transcriptomic datasets of MI patients (GSE66360, GSE62646, GSE19339, GSE62646, GSE61145, and GSE61144) were selected based on established cutoff values. The distributions and numbers of DEGs in each dataset, including up- and downregulated DEGs are presented in volcano plots (Figure 2A). When DEGs in each series were intersected with one another (Figure 2B), seven genes (MAN2A2, TNFRSF12A, SPP1, CSNK1D, PLAUR, PFKFB3, and CXCL16), considered as integrated DEGs (Figure 2C), were obtained. Furthermore, we conducted a meta-analysis of the six databases and identified a total of 4258 and 4754 differentially expressed genes based on the size effect combination and *p*-value combination analysis, respectively (Figure 2). Similar to the DEGs integration method, our meta-analysis also revealed that MAN2A2, TNFRSF12A, SPP1, CSNK1D, PLAUR, PFKFB3, and CXCL16 were significantly over-expressed in all the datasets with effect size combination and *p*-value combination ranges of 5.5~10 zval and 0.0081~1.5 × 10^−9^ fdr_pval respectively (Table 1). The complete results of size effect and *p*-value combination meta-analysis are presented in supplementary file (File S3). Meeting all the cutoff criteria across all the data set analyzed, MAN2A2/TNFRSF12A/SPP1/CSNK1D/PLAUR/PFKFB3/CXCL16 (herein we termed it MTSCPPC signature) was identified as a novel pathological signature of myocardial infarction and was used for subsequent analyses.

### 2.2. Subcellular Localization and Single-Cell Transcriptomic Data of DEGs Signature in the Adult Human Heart during MI

**Figure 3:**
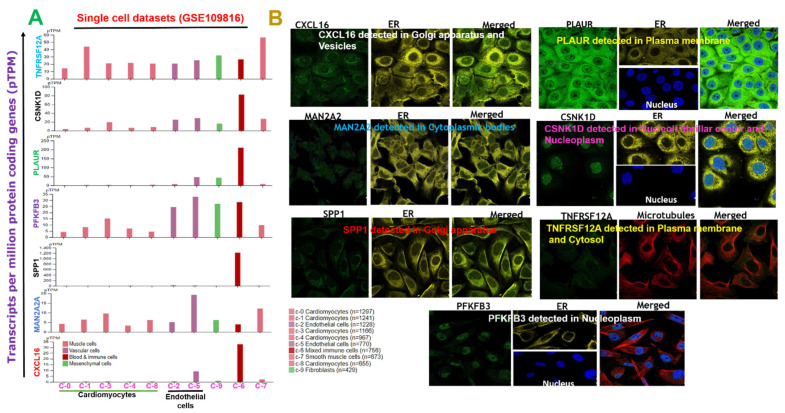
Subcellular localization of proteins and single-cell transcriptomic data of differentially expressed genes (DEGs) in the adult human heart during myocardial infarction. (**A**) Bar plots of single-cell transcriptomic data of the DEGs in different cells during myocardial infarction. (**B**) Immunofluorescence staining of the subcellular distributions of target proteins within the nucleus and endoplasmic reticulum (ER) of cells. Immunofluorescence staining was adopted from the HPA database.

We retrieved single-cell transcriptomic datasets of the adult human heart during MI and compared transcript levels of DEGs in different cells. Our analysis revealed that out of the nine single cells comprising cardiomyocytes, immune cells, endothelial cells, smooth muscle cells, and fibroblasts, expressions of the gene set were highly expressed in immune cells and endothelial cells compared to other single cells of heart tissues during MI (Figure 3A). However, analysis of immunofluorescence (IF) staining from the HPA database for subcellular localizations revealed localization discrepancies of the target genes; PLAUR and TNFRSF12A were localized to plasma membranes, CSNK1D and PFKFB3 were localized in the nucleoplasm, and CXCL16 and SPP1 were localized to the Golgi apparatus (Figure 3B).

### 2.3. The Novel MTSCPPC Signature Is Associated with Disruption of Metabolic and Immune-Inflammatory Pathways during the Pathogenesis of MI

**Figure 4:**
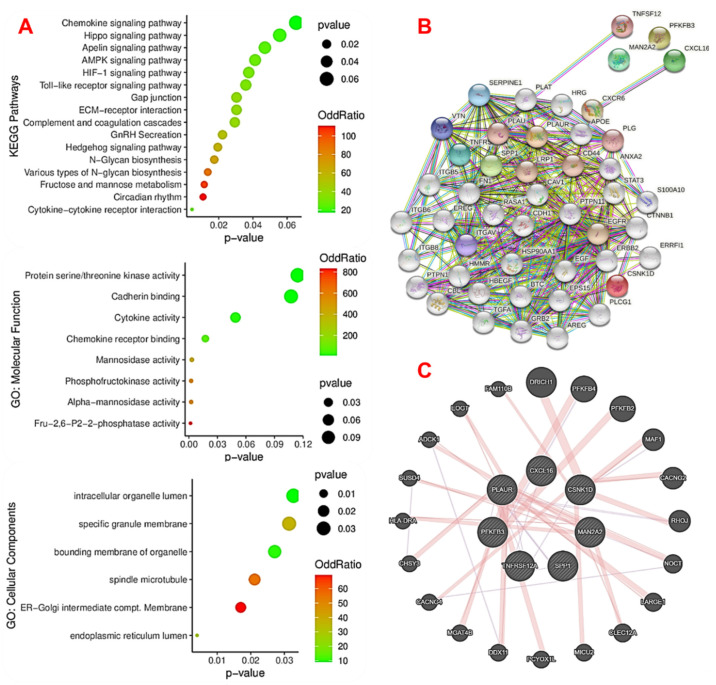
Functional enrichment analysis and biological network analysis of the MTSCPPC signature. (**A**) Visualization of enriched terms in KEGG pathways, and molecular function and cellular component categories in the GO analysis. The color intensity and the size of the round circles indicate, respectively, the odds ratios and *p*-values of enrichment. All terms were only considered to be significantly enriched at *p* < 0.05. Visualization of the significant (**B**) protein–protein interaction (PPI) and (**C**) gene–gene interaction (GGI) network clusters of the DEGs.

To further understand the pathological mechanisms of the DEGs in MI, we conducted functional enrichment and biological network analysis of the MTSCPPC signature. Our results revealed the significant involvement of the MTSCPPC signature in several immune, inflammatory, and metabolic remodelings (Figure 4). In the KEGG pathway analysis, cytokine–cytokine receptor interactions, Toll-like receptor signaling, gonadotropin-releasing hormone (GnRH) secretion, gap junctions, extracellular matrix (ECM)-receptor interactions, complement and coagulation cascades, hypoxia-inducible factor (HIF)-1, adenosine monophosphate-activated protein kinase (AMPK), apelin, Hippo, Hedgehog, chemokine signaling, and various sugar-metabolism pathways were significantly enriched (Figure 4A). Similarly, our GO molecular function enrichment analysis of the MTSCPPC signature identified cytokine activity, chemokine receptor binding, cadherin binding, and activities of various sugar-metabolizing enzymes. The biological process analysis also indicated that several metabolic processes (including carbohydrate, lipid, and protein metabolism pathways), hormonal (androgen and testosterone), and other biological processes (including non-canonical Wnt signaling, fibrinolysis, and T cell chemotaxis) were the most significantly enriched processes of the DEGs (Table 2). To investigate interactions between the proteins encoded by the MTSCPPC signature, PPI and GGI networks were employed (Figure 4B,C). Based on the STRING database, the PPI network was constructed with the MTSCPPC signature, yielding 47 nodes, 359 edges, and a PPI enrichment *p*-value of <1.0 × 10^−16^. The most significantly enriched genes in the PPI included EGFR (23 node degrees), CAV1 (23 node degrees), EGF (22 node degrees), CD44 (22 node degrees), and ANXA2 (19 node degrees) (Figure 4B). On the other hand, the GGI network of the MTSCPPC signature contained 27 human genes (nodes) and 52 interactions (edges, Figure 4C) and generated strict functional enrichment of metabolic pathways associated with carbohydrates, nucleotides, and ATP metabolic processes.

### 2.4. The MTSCPPC Signature Is Implicated in Therapy Resistance and Pathogenesis of Heart Related Diseases

**Figure 5:**
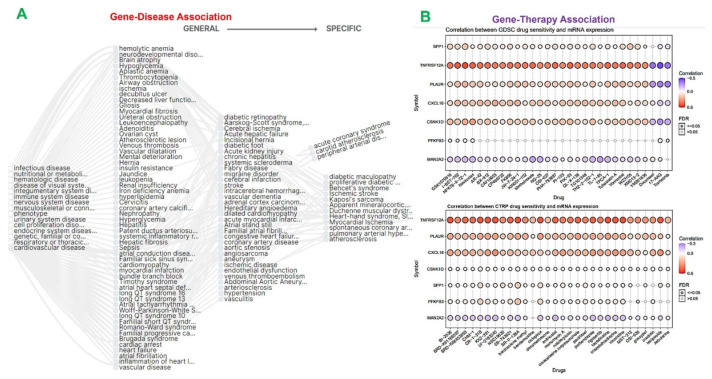
Gene–disease and drug sensitivity associated with the MTSCPPC signature. (**A**) Gene–disease network plot of MAN2A2, TNFRSF12A, SPP1, CSNK1D, PLAUR, PFKFB3, and CXCL16. (**B**) Bubble plot of correlations between gene mRNA expression levels and small-molecule sensitivity. Colors from blue to red represent the correlation between the 50% inhibitory concentration (IC50) value of small molecules and mRNA expression levels of the MTSCPPC signature.

Genomic aberrations are major drivers of disease progression and therapy response. We queried the gene–disease association network of the DEGs. Our analysis suggested the involvement of DEGs in several diseases including ischemic stroke, myocardial ischemia, atherosclerosis, acute coronary syndrome, carotid atherosclerosis, diabetic maculopathy, and so on (Figure 5A). To investigate the roles of the MTSCPPC signature in response to therapy, drug sensitivity and gene expression profiling data were integrated. Our results demonstrated that high expression levels of TNFRSF12A, SPP1, CSNK1D, PLAUR, PFKFB3, and CXCL16 were associated with drug resistance to several small molecules (Figure 5B). However, expression levels of MAN2A2 were associated with increased drug sensitivity. Collectively, our results suggested that the dysregulated expressions of the members of the MTSCPPC signature were associated with the development and progression of several heart-related diseases and could mediate resistance to small molecule drugs.

### 2.5. miRNA Regulatory Network of the DEGs

**Figure 6:**
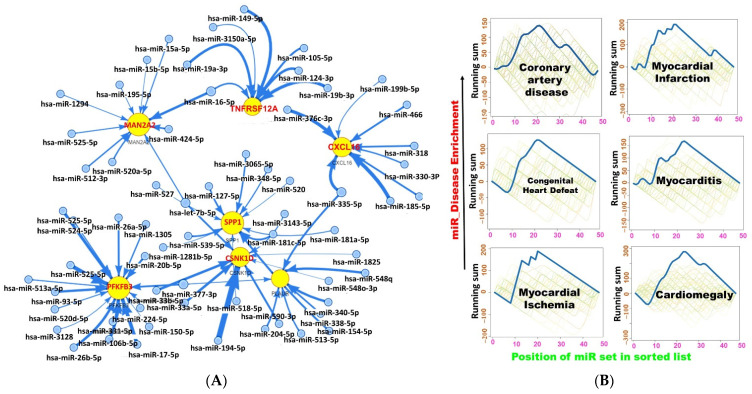
The micro (mi)RNA regulatory network and enrichment of the MTSCPPC signature (**A**) The VisNetwork miRNA regulatory network of the MTSCPPC signature. (**B**) Heart-related disease enrichment plots of the signature.

In order to further understand the mechanisms and pathological roles of the MTSCPPC signature, we queried the miRNA regulation network targets for this signature (Figure 6A). Our analysis revealed that the MAN2A2, TNFRSF12A, SPP1, CSNK1D, PLAUR, PFKFB3, and CXCL16 were regulated by several miRNA regulatory networks involving hsa-miR-149-5p, hsa-miR-3150a-3p, hsa-miR-105-5p, hsa-miR-124-3p, hsa-miR-16-5p, hsa-miR-15a-5p, hsa-miR-15b-5p, hsa-miR-195-5p, hsa-miR-1294, hsa-miR-525-5p, hsa-miR-424-5p, hsa-miR-195-5p, hsa-miR-520a-5p, hsa-miR-512-3p, and several other miRNAs (Figure 6A). Furthermore, we found that the miR regulatory networks were enriched in several pathways, GOs, and disease networks (Figure 6B and Figure S1). Of importance, we found high enrichment of miRNAs in coronary artery disease, MI, myocarditis, congenital heart defects, cardiomegaly, and myocardial ischemia (Figure 6B).

### 2.6. Ovatodiolide, a Macrocyclic Diterpenoid, a Potential Drug for Targeting the MTSCPPC Signature

**Figure 7:**
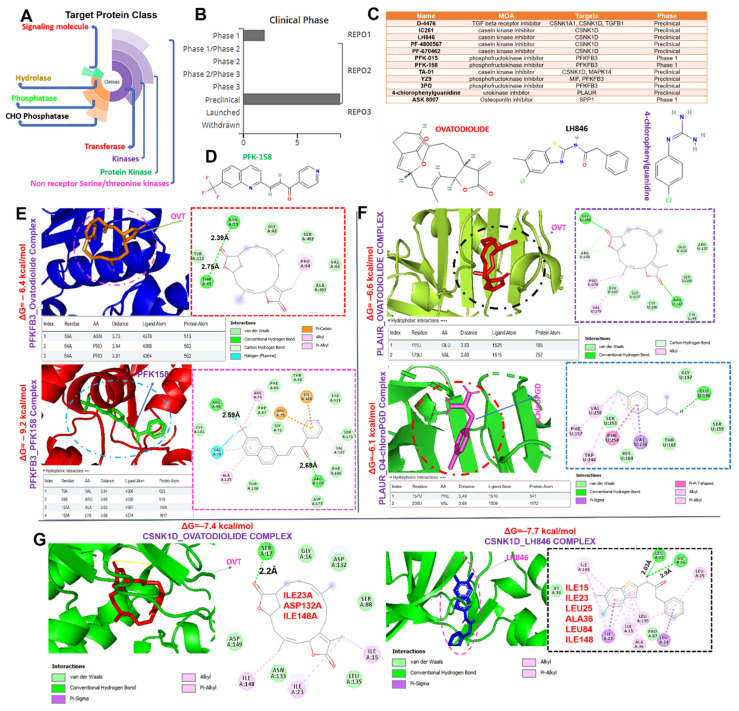
Therapeutic target exploration and comparative docking profile between ovatodiolide and standardized drugs. (**A**) Target protein class, (**B**) clinical phase, (**C**) mechanism of action, and (**D**) chemical structures of validated target inhibitors for the members of the MTSCPPC signature. Three- (3D) and two-dimensional (2D) structures of ligand–receptor complexes, showing interacting amino acid residues and the types of interactions occurring between the ligand (ovatodiolide and standardized drugs) and receptors: (**E**) MAN2A2, (**F**) PLAUR, and (**G**) CSNK1D.

**Figure 8:**
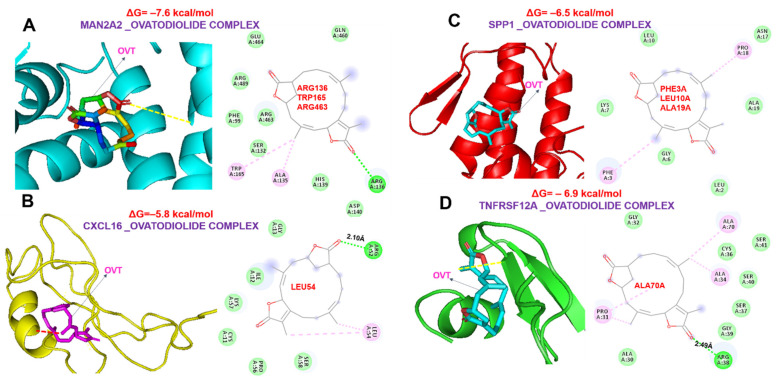
Three- (3D) and two-dimensional (2D) representations of ligand–receptor complexes, showing interacting amino acid residues and the types of interactions occurring between ovatodiolide and (**A**) MAN2A2, (**B**) CXCL16, (**C**) SPP1, and (**D**) TNFRSF12A.

We queried the MTSCPPC signature for the available clinical/preclinical drug targets (Figure 7A–D) and identified several kinase- and phosphatase-targeting small molecules in clinical and preclinical settings. Specifically, two clinical drugs, PFK-015 and PFK-158, for targeting PFKFB3 were identified. The preclinical drugs, including 4-chlorophenylguanidine (PLAUR inhibitor), ASK 8007 (SPP1 inhibitor), LH846, and several other inhibitors of CSNK1D (Figure 7C,D) were identified. Furthermore, we evaluated the therapeutic potential of a natural compound, ovatodiolide, a macrocyclic diterpenoid, by comparing it with the identified clinical/preclinical drugs, its binding affinities, and interaction with amino acid residues of the targets binding sites. Our molecular docking analysis revealed that ovatodiolide docked to the binding cavities of the DEGs with different binding affinities; MAN2A2 (−7.6 kcal/mol), CSNK1D (−7.4 kcal/mol), TNFRSF12A (−6.9 kcal/mol), PLAUR (−6.6 kcal/mol), SPP1 (−6.5 kcal/mol), PFKFB3 (−6.4 kcal/mol), and CXCL16 (−5.8 kcal/mol) (Figure 7 and Figure 8). Several non-covalent interactions, including the H-bonds, halogen bonds, and multiple π-interactions, were found in the complexes formed between the ovatodiolide and the targets (Figure 7 and Figure 8). Furthermore, several hydrophobic contacts and van der Waals forces were found around the ovatodiolide backbone with the respective binding amino acid residues of the receptor’s binding pockets. Judging by the number of interactions and the binding affinities of ovatodiolide, our results suggest that MAN2A2, CSNK1D, and TNFRSF12A are more favored ligands for ovatodiolide therapeutic utility than are PFKFB3, PLAUR, SPP1, and CXCL16. Ovatodiolide demonstrated a higher potential for targeting PLAUR than the preclinical inhibitor of the protein and was comparable to LH846 in its affinity for binding to CSNK1D. However, PFK158, a clinical inhibitor, demonstrated a higher potential for targeting PFKB3 in silico than ovatodiolide. Altogether, our results suggest that ovatodiolide has molecular features for targeting the MTSCPPC signature with higher efficiency for three members of the MTSCPPC signature, namely, MAN2A2, CSNK1D, and TNFRSF12A.

## 3. Discussion

Annual cases of myocardial infarction (MI, acute and chronic) are steadily increasing. Exploring molecular mechanisms of MI is critical to controlling the number of patients with MI and developing precision therapeutic strategies. This study analyzed DEGs from six independent microarray datasets and identified seven overlapping genes (termed the MTSCPPC signature), subsequently analyzed for their pathogenic roles in MI and as therapeutic targets. GO biological and function enrichment analyses identified that the MTSCPPC signature was mainly enriched in several metabolic- (particularly carbohydrate metabolic pathways), hormonal-, immune-, and inflammation-related pathways. Similarly, our KEGG pathway analysis identified cytokine–cytokine receptor interactions, Toll-like receptor signaling, GnRH secretion, gap junctions, ECM-receptor interactions, complement and coagulation cascades, HIF-1, AMPK, Apelin, Hippo, Hedgehog, chemokine signaling, and various sugar-metabolism pathways. Results of these enrichment analyses are in line with higher levels of the MTSCPPC signature in the immune cells than in other cells of the human heart during an MI event, as revealed by our analysis from single-cell transcriptomic databases (Figure 3A). These findings suggest the critical roles of immune and inflammatory responses during MI. Furthermore, the GGI network of the DEGs generated strict functional enrichment of metabolic pathways associated with carbohydrate, nucleotide, and ATP metabolic processes which correlated with our KEGG and ontological findings.

Results of our analysis may provide future directions for better diagnoses and treatments of AMI. Innate immune responses and inflammatory reactions are essential regulators of tissue damage and repair after an MI [39]. Myocardial necrosis induces complement activation, free radical generation, cellular depletion of free radical scavengers, lipid peroxidation, and release of chemotactic factors and triggers a tumor necrosis factor (TNF)-α-mediated cytokine cascade [40,41]. During an MI, immune responses are induced by activating Toll-like receptors on circulating blood cells, increasing the infarct size and mediating ventricular transformation [42]. The chemokine (C-X-C motif) ligand family (Cxcl) contains neutrophil chemoattractants [43], whose overexpression was implicated in aggressive acute inflammation after an MI [44]. Pharmacologic inhibition of Cxcl attenuated MI via neutrophil exclusion at the infarct site [43].

We further investigated interactions between proteins encoded by the MTSCPPC signature via a PPI network and found a significantly higher degree of interactions with EGFR, CAV1, EGF, CD44, and ANXA2. Therefore, we speculated that these genes, which were core genes in the PPI network, might play important roles in regulating cardiac remodeling and be more closely related to MI. Our literature survey revealed that these genes were implicated in immune differentiation, regulation of inflammation, and MI [45,46,47], which indicated that our results of integrated bioinformatics analysis, KEGG, and GO enrichment analysis were reliable. Cav1 was shown to play a critical role in regulating inflammatory responses to an MI by regulating macrophage differentiation in mice [48]. EGF and EGFR were also implicated in the proliferation of cardiac fibroblasts and cardiac remodeling after an MI in rats [47]. CircANXA2 promotes myocardial apoptosis in myocardial ischemia-reperfusion injury [46].

Integrating these genes with the MTSCPPC signature of MI would produce a significantly pronounced pathological phenotype compared to phenotypes of individual genes [49,50]. Per our hypothesis, targeting genes that interact with disease hallmark genes were proposed as a therapeutic concept [51,52] and adopted in clinical trials [53]. Altogether, our study identified the importance of cytokine–cytokine receptor interactions and chemokine signaling, immune and inflammation responses, and metabolic abnormalities in the pathology of MI and identified novel biomarker signature of theranostic relevance for these processes.

Increasing evidence has revealed that dysfunction of 3′- and 5′-untranslated regions (UTRs) of mRNAs is often associated with the pathophysiology of several diseases [54]. miRNAs are small non-coding RNAs that bind to the UTR region and regulate the mRNA translation of target genes [55] and, hence, contribute to the development of various diseases, including MI [56,57]. The binding of miRNAs to the 3′-UTR of target mRNAs is a mechanism (canonical mode) of post-transcriptional repression of target genes [58]. However, recent studies revealed other miRNA regulatory modes that favor post-transcriptional activation [55,59]. We showed that several miRNAs regulated the MTSCPPC signature. These miRNA networks were significantly associated and implicated in coronary artery disease, MI, myocarditis, congenital heart defects, cardiomegaly, and myocardial ischemia (Figure 6B). Our data provided new insights into the molecular mechanism of MI, where miRNA-mediated post-transcriptional activation and regulation of many bioprocesses.

In silico receptor–ligand interaction studies are an important stage in drug discovery and development pipelines. By studying receptor–ligand interactions, behaviors of therapeutic agents around the accommodating cavity of a target protein can be known, and hence we can speculate on the biological activities of drug candidate [21,60] Interestingly, our molecular docking study revealed the potential of ovatodiolide for targeting the MTSCPPC signature in the order of MAN2A2 (−7.6 kcal/mol) > CSNK1D (−7.4 kcal/mol) > TNFRSF12A (−6.9 kcal/mol) > PLAUR (−6.6 kcal/mol) > SPP1 (−6.5 kcal/mol) > PFKFB3 (−6.4 kcal/mol) > CXCL16 (−5.8 kcal/mol). The importance of hydrogen, ionic, hydrophobic, and other non-covalent bonds is crucial to the stability and behavior of a drug candidate within the cavity of a target molecule. The higher affinities of ovatodiolide for MAN2A2, CSNK1D, and TNFRSF12A suggest the differences in therapeutic susceptibilities of the MTSCPPC signature to ovatodiolide. This target favoritism could be attributed to the higher numbers of interactions between amino acid residues of the target proteins and reacting molecules of ovatodiolide. It was reported that high van der Waals forces between a drug candidate and amino acid residues in the binding cavity of the target create a strong, cohesive environment for stabilization of the complexes [60]. Interestingly, ovatodiolide demonstrated a higher potential for targeting PLAUR than the preclinically-validated protein inhibitor and was comparable to the preclinically-validated inhibitor of CSNK1D. However, as expected, PFK158, a clinical inhibitor, demonstrated higher in silico potential for targeting PFKB3 than did ovatodiolide. Collectively, the results from the present study aid our understanding of the complex regulatory mechanisms of MI and provide a promising approach for MI theranostic markers with ovatodiolide as a promising therapeutic candidate.

However, the limitations of the present study merit discussion. The sample size was relatively small as only a few datasets of MI from the GEO database qualified for inclusion. Therefore, larger cohorts of patients with MI are required to confirm the diagnostic relevance of the identified genes. In addition, no validation experiment was performed. In future studies, we plan to perform experiments such as qPCR and Western blot to verify the expression changes of MAN2A2, TNFRSF12A, SPP1, CSNK1D, PLAUR, PFKFB3, and CXCL16, as well as microRNAs that might target the DEGs in experimental models of MI and clinical samples. As we described, the DEGs were associated with metabolic and immune-inflammatory dysregulation. Whether these screened biomarkers are linked to the above indicators in MI patients also requires confirmation. In-depth functional studies including gene over-expressing/silencing and clinical studies such as correlations analysis between gene expression and clinical indicators in MI patients will also be conducted. Furthermore, the full therapeutic potential of ovatodiolide for targeting the DEGs awaits our experimental validation with in vitro and in vivo models.

## 4. Materials and Methods

### 4.1. Transcriptomic Data Acquisition and Identification of DEGs in MI

In total, six datasets (GSE66360, GSE19339, GSE61145, GSE61144, GSE62646, and GSE60993) [61,62,63] consisting of high-throughput gene expression profiles from MI patients and healthy cohorts were downloaded from the NCBI Gene Expression Omnibus (GEO). Detailed information of the microarray datasets is presented in Table 3. Data processing and analysis of DEGs were conducted using the GEOR2 embedded LIMMA package [64] and excel sorting. Analyses were conducted based on the limma contrast selection of all possible pairwise contrasts. The Benjamini–Hochberg correction method was used for *p*-value adjustment of the false discovery rate (FDR) as reported previously [36]. InteractiVenn, a web tool, was used to visualize the intersecting DEGs and generate a Venn diagram to visualize overlapping DEGs [65]. The corresponding raw files from each dataset are presented in supplementary file 1 (File S1). In addition to the conventional DEG integration, we also conducted a meta-analysis of the datasets based on p-value integration and effect size combination methods. The web-based application ImaGEO was used to perform the meta-analysis [66]. The effect size, defined as Z value based on statistical Z-tests, reflects the inter-study variation and the different quantitative measurements used to explain the strength of a phenomenon in different datasets while a p-value reflects whether an effect exists [67]. Therefore, both the substantive significance (effect size) and statistical significance (p-values) are essential for dataset meta-analyses [68]. All our *p*-value analysis was adjusted for multiple testing by the FDR (<0.05) method. All the necessary codes and files to repeat the main analysis are presented in the supplementary section (File S2).

### 4.2. Subcellular Localization and Single-Cell Transcriptomic Data Analysis of the Adult Human Heart during MI

We used the Human Protein Atlas (HPA) database to acquire the subcellular localization of the DEGs based on immunofluorescence staining of the proteins within the nucleus, endoplasmic reticulum (ER), and microtubules [69]. In addition, the single-cell transcriptomic dataset of the adult human heart during heart failure was retrieved from the GEO, with the accession number GSE109816 [70]. The total read counts for genes in each cluster were calculated by adding up the read counts of the genes in all cells belonging to the corresponding cluster. Finally, read counts were normalized to transcripts per million protein-coding genes (pTPM) for each single-cell cluster.

### 4.3. Interaction and Disease Networks, and Gene Set Enrichment Analysis of the DEGs

A DEG enrichment analysis including the Kyoto Encyclopedia of Genes and Genomes (KEGG) pathways and gene ontology (GO) enrichment analyses of the DEGs was conducted using the Enrichr server [71,72], with the enrichment value set to *p* < 0.05. Protein–protein interaction (PPI) enrichment of the DEGs was analyzed using the Multiple Protein modules of the String server [73], while gene–gene interactions (GGIs) of hub genes were evaluated via the GENEMANIA web tool [74]. In order to analyze gene/disease-specific associations of the DEGs, we explored the disease/phenotype-specific filters of the Open Targets Genetics server [75].

### 4.4. Drug Response and Sensitivity Analysis of the DEGs

A drug-sensitivity analysis of the DEGs was conducted through the GSCALite server [76]. We explored Spearman correlation methods to analyze correlations between messenger (m)RNA expression levels of the DEGs and values of the 50% inhibitory concentration (IC50) of small molecules against various cells in the Therapeutics Response Portal (CTRP) and Genomics of Drug Sensitivity (GDSC) databases.

### 4.5. Micro (mi)RNA Regulatory Network and Enrichment Analysis of the DEGs

miRNA regulatory networks of the DEGs were collected from experimentally verified databases (TarBase, mir2disease, and miRTarBase) and predicted databases (miRanda and targetscan). The miRNA regulatory network was visualized using the visNetwork R package. We used the miRNA Enrichment Analysis and Annotation (miEAA) tool to conduct a functional enrichment analysis of sets of miRNA targets [77]. Analyses were conducted using an FDR (Benjamini–Hochberg) adjustment *p*-value of 0.05 and a minimum required hit of four miRNAs.

### 4.6. Comparative Analysis of Ovatodiolide, a Macrocyclic Diterpenoid and Conventional Drugs for Targeting the DEGs

We used the DGIDB database, a drug–gene interaction database [78] and ConnectivityMap, a perturbation-driven gene expression dataset [79] to identify clinical and preclinical drugs for targeting the DEGs. The AutoDock Vina (vers. 0.8, Scripps Research Institute, La Jolla, CA, USA) [80] was used for molecular docking analyses to explore ligand–receptor interactions between target DEGs and drug candidates. All ligand preparations before docking were conducted according to the method described in our previous studies [81,82]. PDB files of the three-dimensional (3D) structures of the target DEGs of MAN2A2 (PDB:1PS3), TNFRSF12A (PDB:2RPJ), SPP1 (PDB:5VFJ), CSNK1D (PDB:4TN6), PLAUR (PDB:6AEX), PFKFB3 (PDB:3QPW), and CXCL16 (PDB:1RJT) were obtained from the Protein Data Bank (PDB). The Avogadro molecular builder and visualization tool version 1.XX [83] was used to build the 3D structure of ovatodiolide in Sybyl mol2 format. SDF files of standard drugs of LH846 (CID: 851474), PFK-158 (CID: 19739459), and 4-chlorophenylguanidine (CID: 2757788) were obtained from Pubchem. All mol2 files were converted to PDB format using the PyMOL Molecular Graphics System, vers. 1.2r3pre, while PDB files were converted to pdbqt format using AutoDock VINA. All dockings were visualized and analyzed with the aid of Discovery studio visualizer (vers. 19.1.0.18287, BIOVIA, San Diego, CA, USA) [84].

## 5. Conclusions

The present study identified MAN2A2/TNFRSF12A/SPP1/CSNK1D/PLAUR/PFKFB3/CXCL16 as a novel and potential signature of cytokine–cytokine receptor interactions, chemokine signaling, immune and inflammatory responses, and metabolic dysregulation associated with MI. Based on the molecular docking of receptor–ligand interaction, ovatodiolide demonstrated the potential for targeting this signature and could be considered for subsequent experimental validation for the development of a new drug.

## Figures and Tables

**Table 1:** Meta-analysis profile of six myocardial infarction (MI) databases.

Gene ID	*p*-Value Combination		Effect Size Combination			Gene_Name
	fdr_pval	FC_mean	fdr_pval	pval	zval	
MAN2A2	0.0005	0.09	7.5 × 10^−7^	2.9 × 10^−8^	5.5	mannosidase alpha class 2A member 2
TNFRSF12A	0.0081	0.13	1.6 × 10^−7^	5.1 × 10^−9^	5.8	TNF receptor superfamily member 12A
SPP1	0.0064	0.33	3.0 × 10^−5^	2.0 × 10^−6^	4.8	secreted phosphoprotein 1
CSNK1D	0.0029	0.13	1.2 × 10^−6^	5.3 × 10^−8^	5.4	casein kinase 1 delta
PLAUR	1.5 × 10^−9^	0.22	0	0	10	plasminogen activator, urokinase receptor
PFKFB3	0.0004	0.13	1.4 × 10^−9^	2.1 × 10^−11^	6.7	6-phosphofructo-2-kinase/fructose-2,6-biphosphatase3
CXCL16	3.0 × 10^−6^	0.17	0	0	8.9	C-X-C motif chemokine ligand 16

Fdr: false discovery rate; FC: fold change; pval: *p*-value; zval: Z value.

**Table 2:** Enriched biological processes associated with the MTSCPPC signature.

Index	Name	*p*-Value	Odds Ratio	Combined Score
GO:0071394	cellular response to testosterone stimulus	0.001749	832.88	5287.72
GO:0006003	fructose 2,6-bisphosphate metabolic process	0.001749	832.88	5287.72
GO:0010818	T-cell chemotaxis	0.003844	333.05	1852.16
GO:0032370	positive regulation of lipid transport	0.004542	277.51	1497.03
GO:0006706	steroid catabolic process	0.004890	256.15	1362.86
GO:2000050	regulation of non-canonical Wnt signaling pathway	0.005239	237.85	1249.08
GO:0042730	fibrinolysis	0.005239	237.85	1249.08
GO:0006491	N-glycan processing	0.006632	184.95	927.70
GO:0045821	positive regulation of glycolytic process	0.007328	166.44	818.24
GO:0008209	androgen metabolic process	0.007328	166.44	818.24
GO:1900544	positive regulation of purine nucleotide metabolic process	0.007328	166.44	818.24
GO:0045913	positive regulation of carbohydrate metabolic process	0.008371	144.71	692.15
GO:0022409	positive regulation of cell-cell adhesion	0.01634	72.27	297.35
GO:0009100	glycoprotein metabolic process	0.01909	61.54	243.60
GO:0031401	positive regulation of protein modification process	0.002310	37.32	226.57
GO:0034341	response to interferon-gamma	0.02767	42.01	150.72
GO:0001934	positive regulation of protein phosphorylation	0.006776	21.27	106.24

**Table 3:** Characteristics of the microarray datasets of myocardial infarction and healthy cohorts.

GEO Accession No.		Platform	Control	MI
GSE60993	GPL6884	Illumina HumanWG-6 v3.0 expression beadchip	7	7
GSE62646	GPL6244	HuGene-1_0-st] Affymetrix Human Gene 1.0 ST Array [transcript (gene) version]2	14	14
GSE19339	GPL570	[HG-U133_Plus_2] Affymetrix Human Genome U133 Plus 2.0 Array	4	4
GSE61145	GPL6884	Illumina HumanWG-6 v3.0 expression beadchip	7	7
GSE61144	GPL6106	Sentrix Human-6 v2 Expression beadchip	10	14
GSE66360	GPL570	[HG-U133_Plus_2] Affymetrix Human Genome U133 Plus 2.0 Array	50	49

## Data Availability

The datasets generated and analyzed in this study can be made available upon reasonable request. http://www.interactivenn.net/ (accessed on 24 August 2021); http://www.ncbi.nlm.nih.gov/geo/ (accessed on 9 August 2021); www.proteinatlas.org; http://string-db.org/ (accessed on 22 August 2021); https://genemania.org/ (accessed on 9 September 2021); https://platform.opentargets.org/ (accessed on 9 September 2021); http://bioinfo.life.hust.edu.cn/web/GSCALite/ (accessed on 5 October 2021); https://ccb-compute2.cs.uni-saarland.de/mieaa2/ (accessed on 28 August 2021); https://www.dgidb.org/interaction; https://clue.io/repg-appurposin (accessed on 1 Septembe 2021).

## Supplementary Materials

The following are available online at https://www.mdpi.com/article/10.3390/ijms23031281/s1.
